# Biocomposites from Organic Solid Wastes Derived Biochars: A Review

**DOI:** 10.3390/ma13183923

**Published:** 2020-09-04

**Authors:** Qingfa Zhang, Hongzhen Cai, Weiming Yi, Hanwu Lei, Haolu Liu, Weihong Wang, Roger Ruan

**Affiliations:** 1 School of Agricultural Engineering and Food Science, Shandong Research Center of Engineering & Technology for Clean Energy, Shandong University of Technology, Zibo 255000, China; qingfa.zhang@wsu.edu (Q.Z.); chzh@sdut.edu.cn (H.C.); 2 Department of Biological Systems Engineering, Washington State University, Richland, WA 99354, USA; 3 Nanjing Institute of Agricultural Mechanization, Ministry of Agriculture and Rural Affairs, Nanjing 210014, China; liuhaolu@caas.cn; 4 Key Laboratory of Bio-based Material Science and Technology (Ministry of Education), Northeast Forestry University, 26 Hexing Road, Harbin 150040, China; Weihongwang2001@nefu.edu.cn; 5 Center for Biorefining and Department of Bioproducts and Biosystems Engineering University of Minnesota, 1390 Eckles Ave., St. Paul, MN 55108, USA; ruanx001@umn.edu

**Keywords:** biocomposites, organic solid waste management, biochar, advances, outlook

## Abstract

The replacement of natural fiber with biochars to prepare biocomposites has attracted widespread attention recently. Biochar has unique properties, including the porous structure, large specific surface area, high thermal stability, good conductivity, renewable and abundant feedstock source, and environmental friendliness, which provide excellent properties, environmental benefits, and low production costs for biochar-based composites. Biocomposites from organic solid waste-derived biochars show good prospects worldwide in terms of positive social, environmental, and economic impacts. This paper reviews current biochars, elucidates the effects of biochars on the characteristics and performance of biochar composites, and points out the challenges and future development prospects of biochar composites.

## 1. Introduction

**Figure 1:**
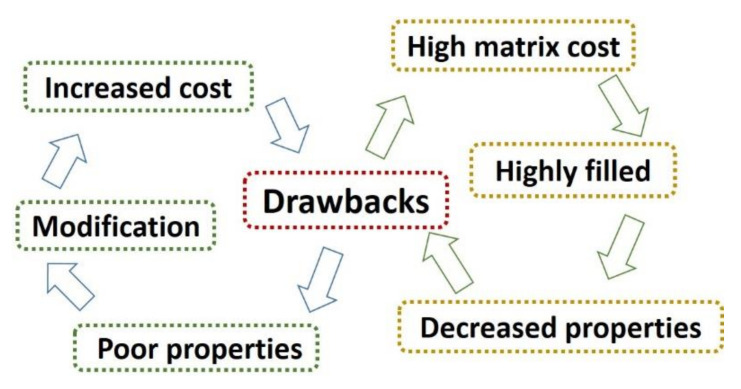
The drawbacks of current biocomposites.

The acceleration of urbanization, industrialization, and population growth rapidly increases the production of organic solid wastes [1], which can be divided into agricultural wastes, forestry wastes, industrial processing wastes, and municipal wastes in terms of sources [2]. Traditional disposal methods including incineration and landfill have disturbed the balance of nature and proved to be unfeasible due to air and water pollution. To overcome this issue, many sustainable methods are studied and used to manage organic solid wastes, including composting [3], fermentation [4], thermochemical treatment [5], and preparing biocomposites [6]. The use of plastic products has improved human life, but the non-degradation of most plastics increases the environmental burden seriously [7]. Based on this, biocomposites derived from organic solid wastes and plastics have exhibited good properties and made great progress over the past decades [8]. Generally speaking, biocomposites are prepared using dried organic solid wastes as fillers and polymeric materials as matrixes by melting and compounding, and this indicates that pollution generation can be reduced or eliminated in the productive process of biocomposites [9,10,11]. Moreover, the biocomposites can be recycled and reutilized in the view of the unique performance of plastics, showing great potential [12]. Although the use of biocomposites displays a sustainable method in managing wastes, there are drawbacks that still cannot be ignored, and the main issues of current biocomposites are shown in Figure 1. Compared to fillers, the cost of the polymeric matrix materials such as polyethylene, polypropylene, etc. is high. Decreasing the proportion of the matrix in the biocomposite system can lower the cost but results in poor properties, especially in injection molding. Requirements for high performance in some special conditions demonstrate the fact that there is an urgent need to improve biocomposite properties. The proportion of fillers and the interface structure are the main factors affecting the properties of biocomposites which need to be improved and have attracted the widespread attention of many scholars [13,14]. Thus, lots of works have been done on improving the interface structure of biocomposites, including filler modification, matrix modification, and adding agents. The main aim of filler modification is to modify the structure and reduce the polarity of fillers, which are currently achieved using heat treatment [15], steam blasting treatment [16], charge-discharge treatment [17], alkali treatment [18], esterification treatment [19], and etherification treatment [20]. These proved to be effective especially for natural plant fiber fillers. In contrast, matrix modifications such as irradiation treatment [21] and oxidation treatment [22] are mainly realized by increasing its surface tension, increasing its surface polarity, and improving its wettability and adhesion. Due to the opposite polarity between the fillers and matrixes, some coupling agents, such as isocyanate, anhydride, and silane, are added as bridges that can effectively link fillers and matrixes. In addition to coupling agents, some other compatibilizers like chlorinated polyethylene [23], methyl methacrylate-butadiene-styrene copolymer [24], and maleic anhydride [25] can decrease the viscosity of the melt and increase the dispersion of fillers in the matrix, thus improving the interfacial compatibility of biocomposites. All the above three modifications are effective in enhancing the interface interactions and improving the properties of biocomposites, but the resulting increase in costs has become another challenge, as shown in Figure 1.

As stated earlier, thermochemical technologies can be considered as one of the effective means to manage organic solid wastes they can create high-value products with no pollution by converting wastes. As the main solid product of thermochemical technologies, biochar is desired for many applications. Recent studies suggest the use of biochars in biocomposites and emphasize minimizing costs and improving properties due to their abundant feedstock and unique structure [26]. Biochar, also named as biocarbon, is usually obtained by gasification or pyrolysis of organic materials under controlled or oxygen-free and high-temperature conditions. All of the organic solid wastes, such as feces, animal bones, agricultural and forestry wastes, can be used as feedstock for producing biochars through a thermochemical process via carbonization or pyrolysis, which can lower the costs for biochar application in biocomposites [27]. The cost of polymer production is much more expensive than biochar due to the complexity of the production processes and the requirements for high-cost raw materials. On the other hand, Bajwa et al. suggested that [28] the coupling agent (e.g., isocyanate, anhydride, etc.) was ineffective for linking polymers and biochars, indicating that the coupling agents in the biochar composite system can be eliminated, which further lowers the costs. After a high-temperature reaction, lots of organic matter is decomposed and volatilized, and most of the functional groups are therefore removed, which contributes to the weak polarity of biochars [29]. The weak polarity of the biochars is beneficial for obtaining a better interface combination with hydrophobic polymeric materials. The porous structure of the biochars can also transfer stress in the biochar composite system, and the mechanical properties, therefore, are improved [30]. In addition to the above advantages, good stability, conductivity, and dispersion are also responsible for the feasibility of biochars in biocomposites [31].

The properties of biochar composites can be affected by the type of biochars, type of polymers, loadings of biochars, and preparation temperatures of the biochars, which indicates that biochar properties play a key role in biochar composites [32,33]. Many research articles have explored the mechanical, thermal, and conductivity properties of biochar composites, but the overview of the manufacture and properties of biochar composites is rarely reported. The aim of this paper is to provide details of the main factors of the properties of biochar composites based on biochar properties, while the ultimate goal is to identify the challenges encountered in biochar composites and evaluate the potential of the conversion from organic solid wastes to high-value products.

## 2. Biochar

Biochar production dates back thousands of years, and the earliest biochar was obtained by incomplete combustion under anoxic conditions using stacked and sealed agroforestry residues such as wood, straw, and so on. The growth in application demands accompanied by technological advances means the traditional biochar preparation process cannot meet current needs. Based on the increasing requirements of biochar yield, efficiency, and quality, new biochar preparation technologies have been developed and continuously innovated. The new reactors and their improvements effectively increased the production yield of biochars. Some typical thermochemical conversion reactors are shown in Table 1. The thermochemical conversion technologies are classified as fast pyrolysis and slow carbonization on the basis of the target products. In order to obtain a high yield of biochars, slow carbonization with a slow heating rate is usually used for biochar production.

### 2.1. Biochar Properties

Biochar is a carbon-rich material with a porous structure and low solubility. Biochar is mainly composed of aromatic hydrocarbons and graphite-like structures of carbon, generally containing more than 60% of the C element, along with other elements such as H, O, N, and S. The aromatic and aliphatic chain structures are important properties of biochars and are the reasons why biochars have strong adsorption and antioxidant capacities [42]. The basic characteristics of biochars can be significantly affected by preparation conditions, including temperature, heating rate, and residence time. Generally speaking, as the preparation temperature increases, the ash content, mineral content, the number of functional groups, the specific surface area, and the alkalinity all increase, but the yield of biochar decreases. In comparison, the increase of the residence time can increase the ash content and biochar yield, but the heating rate lowers the biochar yield [43]. The porous structure, large specific surface area, high carbon content, and stable physicochemical properties are the main contribution to the improvement of biochar composites. The ash in biochars is mainly composed of K, Ca, Mg, Al, Si, etc existing in the form of oxides or salts, which can improve the strength of the polymers acting as rigid particles [44]. The properties of different biochars are shown in Table 2.

### 2.2. Biochar Applications

**Figure 2:**
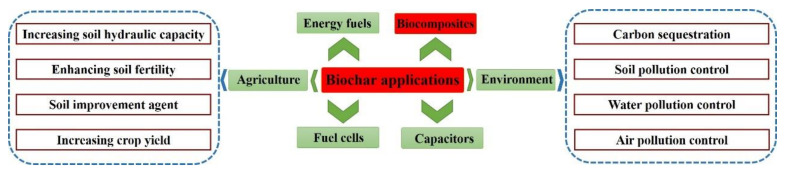
The applications of biochar.

Charcoal became known to humans as early as the Neolithic Age, and its various applications have led to the exploration of its properties, structures, and characteristics. Figure 2 demonstrates the main current applications of biochars. Adding biochar to soil can increase soil hydraulic capacity, enhance soil fertility, and increase crop yield [52]. Great progress has been made in carbon sequestration, soil pollution control, water pollution control, and air pollution control in the environment field [53]. Additionally, biochar also plays a huge role in energy and fuels, fuel cells, and capacitors due to its high calorific value, high strength, large bulk density, flammability, strong stability, and good conductivity [54,55]. Recently, replacing fibers to reinforce polymers using biochars has attracted attention due to the excellent properties and low costs exhibited in biochar biocomposites. Biochar can increase the degradation rate of the polymeric materials in soil, which is of great significance for environmental protection [56]. In 2013, You et al. used charcoal powder to modify ultra-high molecular weight polyethylene (UHMWPE), and 104.7 MPa of tensile strength was obtained in 70% loading of the biochars in composites [57].

## 3. Biochar Composites

**Figure 3:**
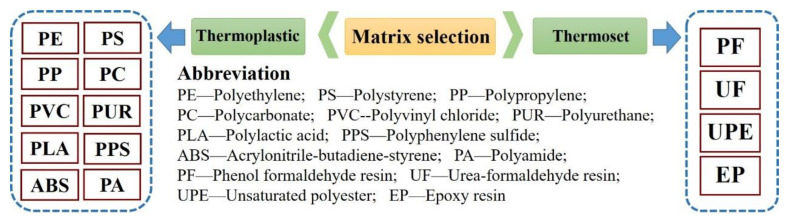
The polymers matrix for biochar composites.

Excellent mechanical and thermal properties are obtained in biochar composites, but high-performance electromagnetic interference shielding and good conductivity are also exhibited due to the unique properties of biochars [58,59]. The fibers are easily degraded at high temperatures (over 200 °C) in natural fiber composites, suggesting limitations via manufacturing temperature, but this issue can be overcome by biochars with good thermal stability [60]. Similar to natural fiber composites, current processes such as extrusion, injection molding, and hot pressing can be used for biochar composites, and the equipment involved are extruders, injection machines, and flat-panel vulcanizers, respectively [61]. As stated earlier, all organic materials can be used to produce biochars as fillers for biochar composites, which demonstrates the advantages of feedstock sources. In addition to fillers, matrix selection also plays a key role in the biocomposite system because polymers can protect the surface of the fillers from abrasion [8]. Some polymers which can be used for biochar composites are shown in Figure 3. Both thermoplastic and thermoset polymers can be used as a matrix in biochar composites due to the good thermal stability of biochars, but the selectivity of thermoset polymers is narrow in terms of their formability, fluidity, and feedstock source.

### 3.1. Interfacial Characteristics

**Figure 4:**
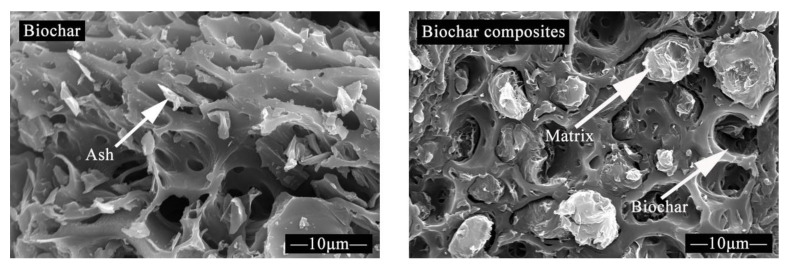
The microstructure of biochar and biochar composites (reproduced with permission from Reference [65]).

The interfacial characteristics, especially the interfacial structure, play an important role in the properties of composites, which are significantly affected by the biochar properties, as stated earlier. Thus, this section discusses the details of interfacial characteristics of biochar composites based on biochar properties, while the ultimate goal is to form the basis for the biochar composite properties. Many documents report that most functional groups were removed after biochar production under heat reaction at high temperatures, and no shifts of polymer peaks were observed with the inclusion of biochars [31,62,63]. This suggested that all the functional groups shown in biochar composites were provided by polymers and no chemical reaction occurred between biochars and polymers. Coincidentally, the lack of polar functional groups, especially hydroxyl, also contributed to a new effect that overcomes the poor compatibility due to different polarities between fibers and polymers in natural fiber composites [26]. Other evidence shows that both the left and right contact angles of biochar composites showed higher values than those of natural fiber composites, indicating the weak hydrophilicity of biochar composites [64]. In the field of composites, X-ray diffraction (XRD) is used to determine the crystal structure of polymer materials, which is also important for characterizing the interface of biochar composites. It is expected that the inclusion of biochars did not change the crystal planes of polymers because of the amorphous nature of biochars, and polymers provided all the diffraction peaks in the biochar composite system. However, the addition of biochar strongly affected the intensity of diffraction peaks, which can be attributed to the loading reduction of crystalline polymers in the composite system [44]. To intuitively characterize the interfacial characteristics of biochar composites, almost all the relevant scholars have explored the microstructure of biochar composites using the scanning electron microscope (SEM), and the results are similar. The microstructure between natural fibers and biochars resulted in the microstructure difference between natural fiber composites and biochar composites. As stated earlier, biochar has a porous structure, and this is the main contributor to the uniqueness of the biochar’s composite structure. In the melting process, thermoplastic polymers melted into fluid at high manufacturing temperatures and were pressed into the biochar pores under equipment pressure, and then the special structure formed after cooling, as shown in Figure 4 [65]. Also, liquid thermosetting polymers were dispersed into the biochar pores under mechanical mixing, and then the special structure was molded after heating [66]. This special structure, named physical/mechanical interlocking [31] by Das et al., can efficiently transfer stress, which is evidence that the biochar composites exhibited great mechanical properties. It has been proven that there is no chemical reaction between biochars and polymers. From this viewpoint, the interfacial interaction of the biochar composites can be summed up in two aspects. The first is the physical/mechanical interlocking. After the polymers fill the pores of the biochar, the biochar engages with the polymers tightly due to the closer contact. Meanwhile, a portion of the residual stress is stored inside the physical/mechanical interlocks, which strengthens the combination of the biochars and polymers. The second should be attributed to the polymers. Polymers acting as binders make a contribution in terms of bonding the biochar and polymer matrix while gumming different biochar particles. More specifically, Van der Waals’ force (VDW) could also provide a solution for the interfacial interaction of biochar composites in terms of molecular attraction. The VDW bond energy is much lower than that of the chemical bond and hydrogen bond, but the large S_BET_ of biochars greatly increased the contact surface between polymers and biochars, which increased the resultant force of numerous VDWs. Hence, the large concentration of numerous VDWs enhanced the interface bonding force [67].

### 3.2. Mechanical Properties 

Biocomposites are widely desired, and the mechanical properties are the key indicators to evaluate for their applications as these reflect the load-bearing capacity of the biocomposites. Among all the mechanical properties, flexural and tensile properties are the most important and frequently tested. From this viewpoint, the mechanical properties of biochar composites have also been tested and reported in many studies. The mechanical properties of biochar composites are reviewed in Table 3. As stated earlier, the loading of biochars in the biochar composite system is the primary factor for mechanical property analysis. There is no significant difference in the mechanical properties of polymers with low biochar loadings. In contrast, appropriate biochar loading can significantly improve the flexural strength, which is mainly attributed to the physical/mechanical interlocking described above and good dispersion of biochars in polymers which efficiently transfers stress [68]. Similarly, the tensile strength of polymers can also be significantly increased via the addition of appropriate biochar loadings. Besides the aforementioned interfacial structure and good dispersion, the good interfacial compatibility formed by the similar polarity between biochars and polymers also contributes to the increment of the tensile strength [69]. Moreover, both the flexural and tensile moduli increased with the incorporation of biochars, as shown in Table 3. These two increases can be attributed to rigidity shown in biochars; the porous and rigid biochar can efficiently reduce the mobility of the polymer matrix when tensile loading is applied to biochar composites [31]. Nevertheless, excessive biochar loadings (over 70%) exhibited an adverse effect on the mechanical properties of biochar composites, the same as natural fiber composites, and the aggregation of excessive biochars in the composite system should be responsible for the decrease of the mechanical properties [30]. The aggregation formed by excessive biochars destroyed the interfacial structure, decreased the interfacial bonding, reduced the plasticity, increased the deformability, and introduced weaker mechanical properties. In addition, it was indicated from previous statements that the basic characteristics of biochars are greatly affected by the preparation conditions, especially the temperature, which means that the biochar preparation temperature is crucial to the interfacial structure and mechanical properties of composites, which can be observed from Table 3. A report by Bartoli et al. suggested that the mechanical properties increased first and then decreased with the increase of biochar preparation temperatures, and the maximum mechanical properties of biochar composites were obtained at 500~600 °C biochar preparation temperatures [70]. Biochar carbonization is incomplete, polarity is relatively high, S_BET_ is relatively small, and the porous structure is poor at low biochar preparation temperatures (below 500 °C), significantly diminishing the interface compatibility and interface structure and thus decreasing the mechanical properties of biochar composites. Further increase in biochar preparation temperatures to 500~600 °C results in the optimal porous structure of biochars and good physical/mechanical interlocking of the biochar composites, which are the main determinants of the best mechanical properties. Although the polarity of biochars is significantly reduced at a range of high temperatures (over 600 °C), the porous structure of biochar is also damaged and collapsed, and the physical/mechanical interlocking of biochar composites is compromised [71]. In summary, the mechanical properties depend on the structure of biochars and biochar composites to a great extent. The types of biochars and matrixes also have been found to affect the mechanical properties. A report by Li et al. suggested that adding biochars into UHMWPE can yield composites with excellent tensile strength over 100 MPa [72]. This is related to the high molecular chain entanglement density, high relative molecular weight, and excellent wear resistance of UHMWPE, in addition to the specific properties of biochars. As for the difference caused by the biochar type, it is mainly affected by biochar strength and rigid inorganic oxides attached to biochars [44]. In addition to improved flexural and tensile properties, the incorporation of biochars can also significantly improve the dynamic mechanical properties (stiffness, elasticity, creep resistance, and anti-stress relaxation ability) of polymers which are better than natural fibers [67,73]. Compared with natural fibers, the porous structure of biochar can hinder the movement of polymer chains and thus improve the dynamic mechanical properties.

### 3.3. Thermal Properties

The good thermal properties of biocomposites are desired for many applications, especially for use in building materials and outdoor decorations. Finding the balance among mechanical properties, thermal properties, and environmental protection is crucial for the development of biocomposites in the future, and biochar composites are no exception to this rule. In order to meet the requirements for high-quality biocomposites, thermal analyses including thermogravimetric analysis (TGA), flame retardant testing, and differential scanning calorimeter (DSC) analysis were used to characterize the thermal properties for the safety design of biochar composites. As mentioned early, the biochar has high thermal stability, with evidence that a relatively smooth thermogravimetric curve can be obtained in biochar indicating no degradation except for water loss due to the increase in temperatures [80]. Good thermal stability is expected to help biochar composites obtain better thermal properties. Many scholars compared the TGA of polymers and the biochar-added composites and observed rather surprising results. The mass-loss rate (DTG) curve exhibited a shift of peaks to higher temperatures in biochar composites in comparison to neat polymer, indicating that the addition of biochars delayed the thermal decomposition rate of polymers, and this is the evidence that the thermal stable biochar improved the thermal properties of polymers [81]. In contrast, a new peak appeared at around 300 °C in the TG curve of natural-fiber-added composites due to the fact that the hemicellulose and cellulose in natural fibers were degraded ahead of time at low temperatures [82]. Thus, another advantage of biochars over natural fibers is that the addition of the natural fiber decreased the thermal properties of polymers. On the other hand, neat polymers have less thermal stability, with the evidence that little residue is yielded in the DTG curve of polymers due to the almost complete degradation. From this viewpoint, the addition of biochars significantly affected the thermal properties of polymers in terms of the high amount of residue produced [31]. Therefore, the thermal stable biochar can efficiently improve the thermal stability in terms of delaying the decomposition rate with increased residue content. As stated earlier, fire resistance is always a crucial factor in the application of biocomposites. Accordingly, cone calorimetry and the limiting oxygen index are used to characterize the fire resistance of biocomposites. The literature has shown that the flammability of natural fiber composites failed to meet the requirements. Although the introduction of flame retardants is proven to be effective, the increase in costs should not be ignored. In the meantime, the fire resistance is also explored and reported in the resultant composites prepared by replacing natural fibers using biochars. The flame-retardant tests by Das et al. [83] and Zhang et al. [44] showed that biochar addition reduced the peak of the heat release and increased the limiting oxygen index of neat polymers, indicating the improvement of fire resistance. The flame-retardant mechanism of the biochars can be analyzed in two aspects. First of all, the thermal-stable biochar in the composite system acted as the insulation barrier and blocked the contact between polymers and air [83]. The other reason can be attributed to the increased ash content produced from biochar preparation. The ash made of nonflammable inorganic matter can be considered as a flame retardant, and it delays the combustion of polymers [84]. Furthermore, DSC could also provide other evidence for the better thermal properties of biochar composites via their melting and crystallization behavior. Studies by Li et al. and Pan et al. have shown that a slight decrease in melting temperature can be observed in biochar composites compared to neat polymers, and this suggested that the inclusion of biochars decreased the lamella thickness and crystals [67,85]. The interesting result is the exothermic crystallization which occurred in the cooling phase. Adding biochars significantly increased the crystallization temperature of polymers, indicating that the addition of biochars promoted the crystallization of polymers, which is important for shortening the molding cycle of the biochar composites. Biochars acted as points from where crystal growth was initiated, and the nucleation effect of them accelerated the crystallization of polymers in composites [69,86], which is similar to the effect of natural fiber particles. Besides, it can be seen in Table 4 that the crystallinity of biochar composites is also reduced after the incorporation of biochars, and this reduction can be attributed to the structure of the biochars. The pores of biochars restrict the movement of polymer chains and hinder the transition of polymer segments to their crystal form [74].

### 3.4. Electrical Properties

Natural fiber composites are notoriously non-conducive due to the non-conductive properties of polymers and natural fibers, which are unfavorable for composite functionalization. Table 5 demonstrates a review of conductivity in biochar composites. The biochar is rich in carbon with highly stable physicochemical properties. The increase of aromatization and graphitization is achieved with increasing biochar preparation temperatures [91]. These properties give biochar good conductivity, and the introduction of biochars to polymers is expected to provide the possibility of preparing conductive composites. Lots of studies have been done to explore the electrical properties of biochar composites, as illustrated in Table 5. Nan et al. [92] studied the electrical response behavior of poly (vinyl alcohol) (PVA)/wood-derived biochar composites. They found that the increase of biochar loadings improved the conductivity and significantly decreased the electrical resistance of PVA/biochar composites. The increasing biochar content contributed to the conductive paths in the composite system. Electromagnetic interference shielding (EMISE) is used to measure the barrier effect of materials on electromagnetic waves and characterize electrical properties. Surprisingly, 10 dB and 48.7 dB of EMISE were obtained in 20% biochar composites and 80% biochar composites reported by Savi et al. [93] and Li et al. [58], respectively, which indicates that biochars facilitated the excellent EMISE performance in the composite system. This can be attributed to the conversion and dissipation of the electromagnetic wave energy to electricity and heat energy due to the good conductivity, porous structure, and large S_BET_ of biochars [94]. The conductivity of biochar composites is mainly determined by two factors, i.e., biochar preparation temperatures and biochar loadings, as shown in Table 5. The conductivity increases as these two things increase. Increasing the preparation temperatures resulted in the enlargement of the aromatic region and increased the conductivity of biochars [76,95]. Increasing biochar loadings can facilitate the conductive chains and conductive clusters inside composites [96,97]. Biochar composites compensated for the defects of the electrical properties of natural fiber composites, which is crucial for the wide application of biocomposites. 

## 4. Conclusion and Outlook

This paper gives an overview of biocomposites made from biochars that are produced from organic solid wastes. The interfacial characteristics and typical properties of biochar composites are analyzed and discussed. Compared with natural fiber composites, biochar composites exhibited better mechanical, thermal, and electrical properties due to the special nature, i.e., porous structure, large specific surface area, high thermal stability, and good conductivity of biochars. Moreover, the abundance of biochar feedstock sources and the removal of additives in composites can significantly lower the costs of biochar composites. The balance achieved by the properties and costs justifies the renewability and sustainability of the biocomposites, which is crucial for their wide applications across many areas. Nevertheless, further development is required for biochar composites to meet the great challenges of biocomposites and their applications.

**Figure 5:**
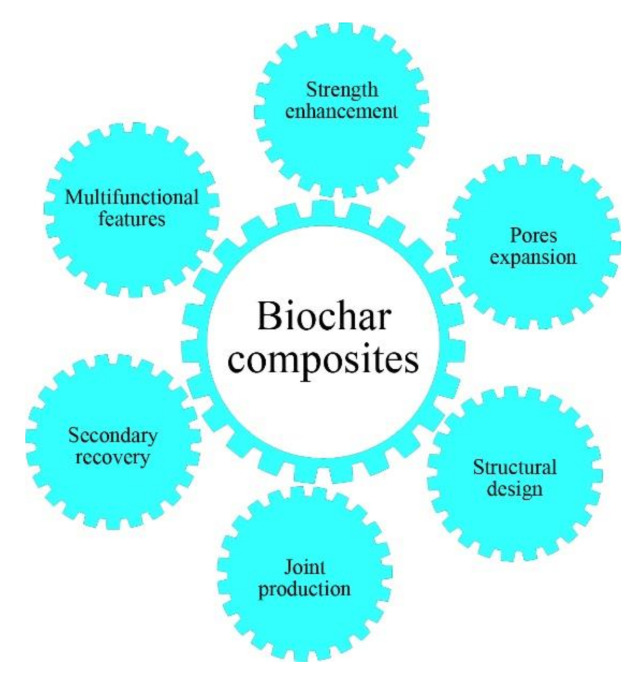
Suggestions for the future development of biochar composites.

Biochar composites have excellent mechanical, thermal, and electrical properties with good water and wear resistance which paves the way for their use. Other properties, especially aging resistance, weather resistance, antibacterial, and sound absorption, are also desired for many biocomposites. As for the outlook of biochar composites, some suggestions are made and shown in Figure 5. The multifunctionalization of biocomposites must be studied and developed to respond to the growing requirements for various applications. Meanwhile, high-strength biocomposites are required in some specific applications, and there is a need to enhance the strength of biochar composites. The addition of rigid particles such as inorganic oxide, carbon fiber, mineral fiber, synthetic fiber, and so on can be considered and studied, and the synergy mechanisms between biochars and rigid particles should also be considered, as they are important for the strength enhancement of biochar composites. As stated above, the porous structure and large specific surface area are the main reasons for the good mechanical properties. From this viewpoint, further expansion of the biochar structure and specific surface area via procedures such as activation can be another target for establishing a correlation between the pore characteristics of biochars and the mechanical properties of biochar composites. Additionally, the structural design of biochar composites is a key factor to be considered in practical applications, and the future development of biochar composites should focus on performance and structure integration design to meet social needs for material design. Furthermore, the premise of biochar composite development and application is to improve production efficiency, which requires cooperation between the production system and production equipment. Many factors, i.e., the storage and transportation of organic solid wastes, large scale carbonization equipment, and composites workshops should be considered in detail. The final thought is the recovery of biochar composites, and this is crucial in the production life-cycle system. The specifics of polymers and biochars yield the recyclability of biochar composites, which achieve a balance in terms of protecting the environment and conserving resources. Overall, the future development potential of biochar composites is very positive, with new designs anticipated to solve significant challenges.

## Figures and Tables

**Table 1:** Typical thermochemical conversion reactors.

Reactors	Advantages	Disadvantages	References
Fluidized bed reactor	Simple construction, operation and high efficiency of heat transfer	High operation cost	[34]
Vacuum moving bed reactor	Short residence time	Lower transfer rate	[35]
Auger reactor	Less complex and low cost	Lower liquid yield	[36]
Rotating cone reactor	Using larger particles	Less effective scaling	[37]
Down-tube reactor	High heat transfer rate and short residence time	Long heat transfer stroke	[38]
Fixed bed reactor	Simple structure	Poor sealing	[39]
Microwave reactor	Easy control and energy saving	Lower capacity	[40]
Muffle furnace	Energy saving and simple construction	Lower capacity	[41]

**Table 2:** The main properties of several typical biochars.

Biochars	Temperatures (°C)	Yield (%)	S_BET_ (m^2^/g)	C	O	H	N	References
Corn straw biochar	400	53.94	7.15	75.14	18.92	4.56	1.38	[45]
Wheat straw biochar	500	28.3	7.4	76.4	19.5	3.4	0.7	[46]
Pine saw dust biochar	550		431.91	59.19	20.73	3.97	0.51	[47]
Rice husk biochar	450		18.58	46.56	18.58	3.54	0.85	[47]
Swine manure biochar	500	40.9		33.8	14.1	2.39	2.23	[48]
Tire biochar	600	37.6		66.6	21.1	0.21	0.09	[48]
Bamboo biochar	600		181.05	82.92	5.03	2.19	0.49	[49]
Grape marc biochar	500	33.8	205	72.91	12.9	3.15	2.72	[50]
Palm kernel shell biochar	750	31.15	394.53	78.95	18.27	1.79	1.00	[51]

**Table 3:** The mechanical properties of some biochar composites.

Biochars	Polymers	Biochar Temperature (°C)	Biochar Loading (%)	Flexural Strength (MPa)	Flexural Modulus (GPa)	Tensile Strength (MPa)	Tensile Modulus (GPa)	References
Date palm biochar	PP	900	15			34	1.36	[74]
Pine wood biochar	PP	900	36	59	3.2	31	3.3	[68]
Maple tree biochar	EP	1000	20			16	0.7	[66]
Rice husk biochar	HDPE	600	50	34.95	1.76	26.25	1.87	[44]
Switchgrass biochar	PLA	500	20	60	3.4	54	1.9	[28]
Switchgrass biochar	HDPE	500	20	12	0.8	23	0.6	[28]
Nano bamboo biochar	UHMWPE	1000	9			24.7	0.36	[72]
Pine wood biochar	PP	900	30	59	3	29	3.48	[31]
Miscanthus biochar	PC/PFA/EP	500	20	113		57.9	3.2	[75]
Coffee biochar	EP	1000	15			25	3.26	[76]
Wasted cotton biochar	EP	400	5			23	1.6	[77]
Charcoal	UHMWPE	500	70			102		[78]
Bamboo biochar	UHMWPE/LLDPE	1100	80			28.4	1.18	[58]
Miscanthus biochar	Nylon 6	500	20			97	3.15	[79]
Bamboo biochar	PLA		7.5	38.98	0.76	51	3.7	[69]
Olive trunks biochar	EP	400	15			17	1.4	[70]
Thuja occidentalis biochar	PP	700	10	62	2.4	32.3	2.5	[62]

Acronyms: PP—polypropylene; EP—epoxy resin; HDPE—high-density polyethylene; PLA—polylactic acid; UHMWPE—ultra-high molecular weight polyethylene; PC—polycarbonate; PFA—poly(furfuryl alcohol); LLDPE—linear low-density polyethylene.

**Table 4:** The melting and crystallization behavior of some biochar composites.

Samples	Biochar Loading (%)	T_m_ (°C)	T_c_ (°C)	X_c_ (%)	References
PP Date palm biochar added	0 5	166.78 164.4	120.97 121.34	43.0 33.42	[74]
Nylon Miscanthus biochar added	0 20	217.9 217.2	196.4 194.9	32.67 30.04	[79]
PLA Bamboo biochar added	0 7.5	159.84 156.68	129.09 135.11	49.50 13.90	[69]
PLA Ultrafine bamboo-char added	0 40	149.7 142.2	126.3 94.4	1.09 22.06	[87]
PLA Coffee biochar added	0 2.5	169.5 168.3	102.5 96.9	13.9 23.3	[88]
PVA Hard wood biochar	0 10	214.96 192.82	290.11 337.10	57.70 41.88	[89]
PP Tea leaves biochar added	0 30	165 165	117 128	53 53	[90]
UHMWPE Bamboo biochar added	0 80	135.1 132.8	119.6 122.8		[67]
HDPE Poplar biochar added	0 70	131 130	120 123		[63]

Acronyms: PP—polypropylene; PLA—polylactic acid; PVA—polyvinyl alcohol; UHMWPE—ultra-high molecular weight polyethylene; HDPE—high-density polyethylene; T_m_—melting temperature; T_c_—crystallization temperature; X_c_—crystallinity.

**Table 5:** Conductivity of some biochar composites.

Composites Samples	Biochar Temperature (°C)	Biochar Loading (%)	Conductivity (S/cm)	References
Bamboo biochar/UHMWPE	1000	7	1.1 × 10^−2^	[72]
Coffee biochar/EP	600	5	2	[76]
Coffee biochar/EP	1000	20	2.02 × 10^2^	[76]
Pine biochar/UHMWPE	1100	50	2 × 10^−1^	[78]
Plastic waste biochar/EP	450	5	6.54 × 10^−8^	[97]
Pine cone biochar/EP	450	25	6.07 × 10^−3^	[97]
Maple wood biochar/EP	950	20	1.3 × 10^1^	[27]
Apple biochar/UHMWPE	700	70	1.7 × 10^−3^	[29]
Apple biochar/UHMWPE	900	70	8.2 × 10^−2^	[29]
Miscanthus biochar/EP	650	20	2 × 10^1^	[59]
Miscanthus biochar/EP	750	20	2.75 × 10^2^	[59]
PET biochar/oligomers	450	50	1 × 10^−2^	[98]

Acronyms: UHMWPE—ultra-high molecular weight polyethylene; EP—epoxy resin; PET—polyethylene terephthalate.
